# Childhood malnutrition, rickets, and anemia: a systematic review and meta-analysis on global prevalence, determinants, and public health implications

**DOI:** 10.3389/fpubh.2026.1786959

**Published:** 2026-03-16

**Authors:** Yajuan Tan

**Affiliations:** Traditional Chinese Medicine Pediatrics, The Central Hospital of Enshi Tujia and Miao Autonomous Prefecture, Enshi, China

**Keywords:** calcium deficiency, childhood malnutrition, global nutrition, maternal nutrition, micronutrient supplementation, nutritional rickets, public health, vitamin D deficiency

## Abstract

**Background:**

The world continues to face major health risks through childhood malnutrition and rickets and anemia which hinder the development of physical and mental and immune system functions. The public health system needs to study determinants of health problems and their effective solutions.

**Methods:**

Our research team evaluated 96 studies, which include 153,694 participants from the Middle East and Africa and South and Southeast Asia and high-latitude regions through systematic review. The research team searched for studies in PubMed, Web of Science, Embase, Cochrane Library, and Google Scholar. The research team included observational studies and RCTs and cohort studies and individual participant data meta-analyses in their investigation. The study team evaluated research quality and bias risk through the use of Jadad scale and GRADE system and standard evaluation tools. The research team conducted random-effects meta-analyses to combine hazard ratio data while they used funnel plots and Egger’s test to check for publication bias.

**Results:**

The study found that Childhood Nutritional Rickets and Vitamin D/Calcium Status showed strong associations with low calcium and vitamin D intake (HR 1.51, 95% CI 1.26–1.82; I^2^ = 88%). The research team found that three programs which included Childhood Malnutrition Prevention and Micronutrient Supplementation and Maternal/Early Childhood Nutrition Programs showed protective effects (HR 0.80, 95% CI 0.77–0.84; I^2^ = 0%; HR 0.91, 95% CI 0.86–0.96; I^2^ = 22%; HR 0.85, 95% CI 0.78–0.93; I^2^ = 53%). The research team used long-term observational studies to demonstrate persistent malnutrition without significant pooled effects (HR 0.96, 95% CI 0.90–1.01; I^2^ = 10%). Publication bias was found in Groups 1–4 (Egger’s *p* < 0.001–0.001), publication bias was not found in Group 5 (*p* = 0.054).

**Conclusion:**

The combination of calcium and vitamin D supplements with targeted micronutrients and integrated maternal-child programs effectively decreases rickets and malnutrition while it enhances global growth rate. The implementation of programs requires specific contextual understanding for achieving the best health results in children.

## 1 Introduction

The ongoing battle against childhood malnutrition constitutes a major public health crisis which will persist throughout the 21st century because it disrupts the natural process of growth and development and threatens the lives of millions of children across the globe. The world continues to experience malnutrition problems which show various complex patterns that include both undernutrition and micronutrient deficiencies as well as hidden metabolic disorders which create severe health problems for people and their communities. The two nutritional rickets and anemia demonstrate high occurrence rates because they stem from multiple sources which together create permanent damage to both physical and cognitive abilities of those who develop these conditions. This systematic review and meta-analysis seeks to quantify the global burden of these conditions, explore their determinants, and evaluate the implications for public health policies and intervention strategies (1–4).

Malnutrition occurs during early life when people fail to consume or absorb the necessary macronutrients and micronutrients needed for their growth and bodily functions. The World Health Organization (WHO) states that undernutrition causes approximately 45% of fatalities which occur in children younger than five years in low-and middle-income countries (LMICs) that experience food shortages and financial hardship and have restricted medical services. The 2020 Global Nutrition Report estimated that more than 149 million children under five years of age experience stunting while 45 million children experience wasting which represents the two types of malnutrition. The conditions make people more vulnerable to infectious diseases because they harm their immune systems and reduce their ability to think which creates a cycle of poor health together with economic hardship (5–9).

The nutritional rickets condition represents a reversible yet uncommon disorder which leads to impaired bone mineralization in children who suffer from malnutrition. Rickets now affects patients who experience vitamin D deficiency and who do not consume enough calcium through their diet and who experience various health effects from their sun exposure and their dietary patterns and their genetic traits and their cultural backgrounds. The condition manifests through multiple clinical symptoms which include bone pain and deformities and delayed growth and higher susceptibility to fractures. The renewed occurrence of rickets across various geographical areas demonstrates that both developing nations and high-income countries with their vulnerable groups need better preventive methods which include vitamin D supplementation and food fortification and public health educational initiatives. The existing clinical surveillance data shows regional prevalence information but researchers have not established complete worldwide rickets prevalence data which includes its causes thus making it difficult to create effective policies (10–13).

Anemia exists as a medical condition that causes decreased blood hemoglobin levels and leads to problems with oxygen distribution throughout the body which develops together with malnutrition and rickets. Anemia during childhood affects approximately 39% of children between the ages of 6 to 59 months who live in various parts of the world with sub-Saharan Africa and South Asia showing the highest prevalence rates. The origins of anemia result from multiple factors which include iron deficiency as the primary cause and vitamin A folate vitamin B12 deficiencies and chronic infections and inflammation and inherited hemoglobin disorders. Anemia during early childhood leads to three major consequences which include decreased energy levels and restricted ability to think and move and higher chances of death. The combination of anemia and other forms of malnutrition which includes stunting and micronutrient deficiencies creates greater risks for development while making it harder to treat patients and organize public health initiatives (14–22).

The research findings show that these medical conditions cause substantial health problems which lead to children’s death rates in various locations but show different rates which develop various health problems for different groups of people. The epidemiological situation shows complicated patterns which arise from diverse dietary habits and cultural traditions that dictate sun exposure and different income levels and available medical services. The treatment of rickets and anemia requires combined research because the two conditions together with all other types of malnutrition need complete examination for development of successful treatment methods. New research from controlled trials and observational studies shows that various dietary programs which contain both micronutrient supplements and additional food options and cash-based conditions can help decrease the likelihood and intensity of malnutrition along with its related health effects. The methods cannot be effectively applied to different communities until scientists establish strong meta-analytic proof of their effectiveness (23–32).

This systematic review and meta-analysis therefore aims to bridge the evidence gap by providing updated global estimates of the prevalence of childhood malnutrition, rickets, and anemia; identifying key determinants and contextual factors; and evaluating the implications for public health policy and implementation. The study collects different population data and various research methods to create guidelines which help policymakers and clinicians and program planners decide which prevention methods and early detection techniques and integrated management systems to implement. The global burden of interrelated conditions requires understanding their distribution because this knowledge supports the development of interventions which help children reach their maximum growth potential while achieving better health outcomes throughout their lives.

## 2 Methodology

### 2.1 Study design and reporting standards

The researchers conducted this systematic review and meta-analysis to assess three factors which are the worldwide occurrences and the causes and the effects of childhood malnutrition and rickets and anemia on public health. The study followed the Preferred Reporting Items for Systematic Reviews and Meta-Analyses (PRISMA) guidelines to ensure methodological transparency, reproducibility, and comprehensive reporting. This is a primary meta-analysis of individual research studies.

### 2.2 Search strategy

The researcher conducted a comprehensive literature search across all major electronic databases which included PubMed, Web of Science, Embase, Cochrane Library, and Google Scholar to access all published materials from the inception of the databases to their most current content. The research team performed a manual search of reference lists found in relevant articles and earlier published reviews to guarantee that they completed a comprehensive search. The search strategy combined controlled vocabulary such as Medical Subject Headings (MeSH) with free-text keywords using Boolean operators (AND, OR). The key terms which researchers used for their study included “childhood malnutrition” and “undernutrition” and “nutritional rickets” and “vitamin D deficiency” and “calcium deficiency” and “anemia” and “iron deficiency” and “micronutrient deficiency” and “prevalence” and “risk factors” and “children.” The researchers established no geographic boundaries because they wanted to collect evidence from every part of the world (Table 1).

**Table 1:** Full search strategies for all databases.

Database	Search strategy	Filters/Limits
PubMed	(“child*” OR “infant*” OR “toddler*” OR “preschool*”) AND (“malnutrition” OR “undernutrition” OR “stunting” OR “wasting” OR “underweight”) AND (“rickets” OR “vitamin D deficiency” OR “calcium deficiency”) AND (“anemia” OR “iron deficiency” OR “hemoglobin” OR “hematologic disorders”)	Humans, all languages, from inception to Dec 2025
Embase	(‘child’/exp. OR ‘infant’/exp. OR child* OR infant* OR toddler*) AND (‘malnutrition’/exp. OR ‘undernutrition’/exp. OR malnutrition OR undernutrition OR stunting OR wasting OR underweight) AND (‘rickets’/exp. OR rickets OR ‘vitamin D deficiency’ OR ‘calcium deficiency’) AND (‘anemia’/exp. OR anemia OR ‘iron deficiency’ OR hemoglobin)	Human studies, all years, all languages
Web of Science	TS = (child* OR infant* OR toddler* OR preschool*) AND TS = (malnutrition OR undernutrition OR stunting OR wasting OR underweight) AND TS = (rickets OR “vitamin D deficiency” OR “calcium deficiency”) AND TS = (anemia OR “iron deficiency” OR hemoglobin)	All document types, no language restrictions
Cochrane Library	(“child*” OR “infant*” OR “toddler*”) AND (“malnutrition” OR “stunting” OR “wasting” OR “underweight”) AND (“rickets” OR “vitamin D deficiency” OR “calcium deficiency”) AND (“anemia” OR “iron deficiency”)	Cochrane reviews and trials, all years
Google Scholar	“child*” OR “infant*” OR “toddler*” AND malnutrition OR undernutrition OR stunting OR wasting OR underweight AND rickets OR “vitamin D deficiency” OR “calcium deficiency” AND anemia OR “iron deficiency”	First 200 results per search, all years, all languages

### 2.3 Eligibility criteria

The researcher employed predefined rules which included specific requirements and particular exclusions to select their studies according to the Population-Intervention/Exposure-Comparison-Outcomes-Study Design (PICOS) framework. The research qualified studies which examined nutritional problems through malnutrition and rickets and anemia studies with Child and Adolescent participants from birth until age 18. The research included observational studies which used cross-sectional and cohort and case–control methodologies to evaluate disease patterns while using randomized controlled trials to track nutritional treatment results. The research accepted only peer-reviewed studies which provided enough numerical information for researchers to extract. The research excluded all forms of case reports and editorials and conference abstracts and narrative reviews and adult-only studies and articles without essential outcome data. The research eliminated all non-English documents.

### 2.4 Study selection process

We imported all retrieved records into reference management software and used the software to eliminate duplicate records before they started their screening process. The reviewer evaluated titles and abstracts to identify potentially relevant studies. The author evaluated full-text articles to check their compliance with pre-established eligibility standards. The reviewer handled their conflicts through discussion while they needed outside help from a third reviewer to achieve agreement. The study selection process used a PRISMA flow diagram which provided complete documentation of the entire procedure for better visibility. For studies reporting multiple relevant outcomes, we prioritized clinically primary outcomes first, followed by secondary outcomes, to avoid duplication.

### 2.5 Data extraction

The researchers created a standardized data extraction form which enables them to collect essential information from each research study in a uniform manner. The researchers gathered data which included the first author name, publication year, study country, research design, participant count, participant characteristics, diagnostic criteria for malnutrition, rickets, and anemia, disease prevalence data, related risk factors, details about the interventions that were used, and major outcome measurements. The reviewer conducted independent data extraction which they followed by cross-checking to achieve greater precision in results. Effect measures were harmonized: odds ratios (ORs) were converted to hazard ratios (HRs) where appropriate; continuous outcomes were standardized to common units. Missing data were handled by exclusion when non-recoverable, and where feasible, imputation methods were applied.

### 2.6 Quality assessment and risk of bias

The researchers used validated tools for evaluating and grading the methodological quality of the studies they included. The Jadad scale assesses randomized controlled trials by measuring their randomization methods and blinding techniques and withdrawal reporting methods. The Newcastle–Ottawa Scale (NOS) provides established risk-of-bias assessment frameworks for observational studies which evaluate selection methods and group comparability and outcome assessment. The Grading of Recommendations Assessment Development and Evaluation (GRADE) method assesses evidence certainty for primary outcomes through four evidence levels which depend on study limitations and result consistency and measurement accuracy and study directness likelihood.

### 2.7 Statistical analysis

The researchers used dedicated statistical software to conduct quantitative synthesis, which generated combined effect sizes and prevalence rates together with their 95% confidence interval results. The researchers implemented a random-effects model because they expected the studies to exhibit both clinical and methodological differences. The researchers used Cochran’s Q test to assess statistical heterogeneity which they measured through I^2^ statistic values that showed low heterogeneity at 25% moderate heterogeneity at 50% and high heterogeneity at 75%. The researchers conducted subgroup and sensitivity analyses to investigate the effects of different geographic regions study designs age groups and nutritional deficiency types when they found major heterogeneity in their results. The researcher evaluated publication bias by analyzing funnel plots visually and Egger’s test.

### 2.8 Ethical considerations

The review required no ethical approval because it used data from studies that had already been published. The researchers conducted their study according to established ethical guidelines for secondary data analysis which involved them maintaining accurate reporting and proper citation practices while interpreting their findings.

## 3 Results

### 3.1 Study selection

**Figure 1:**
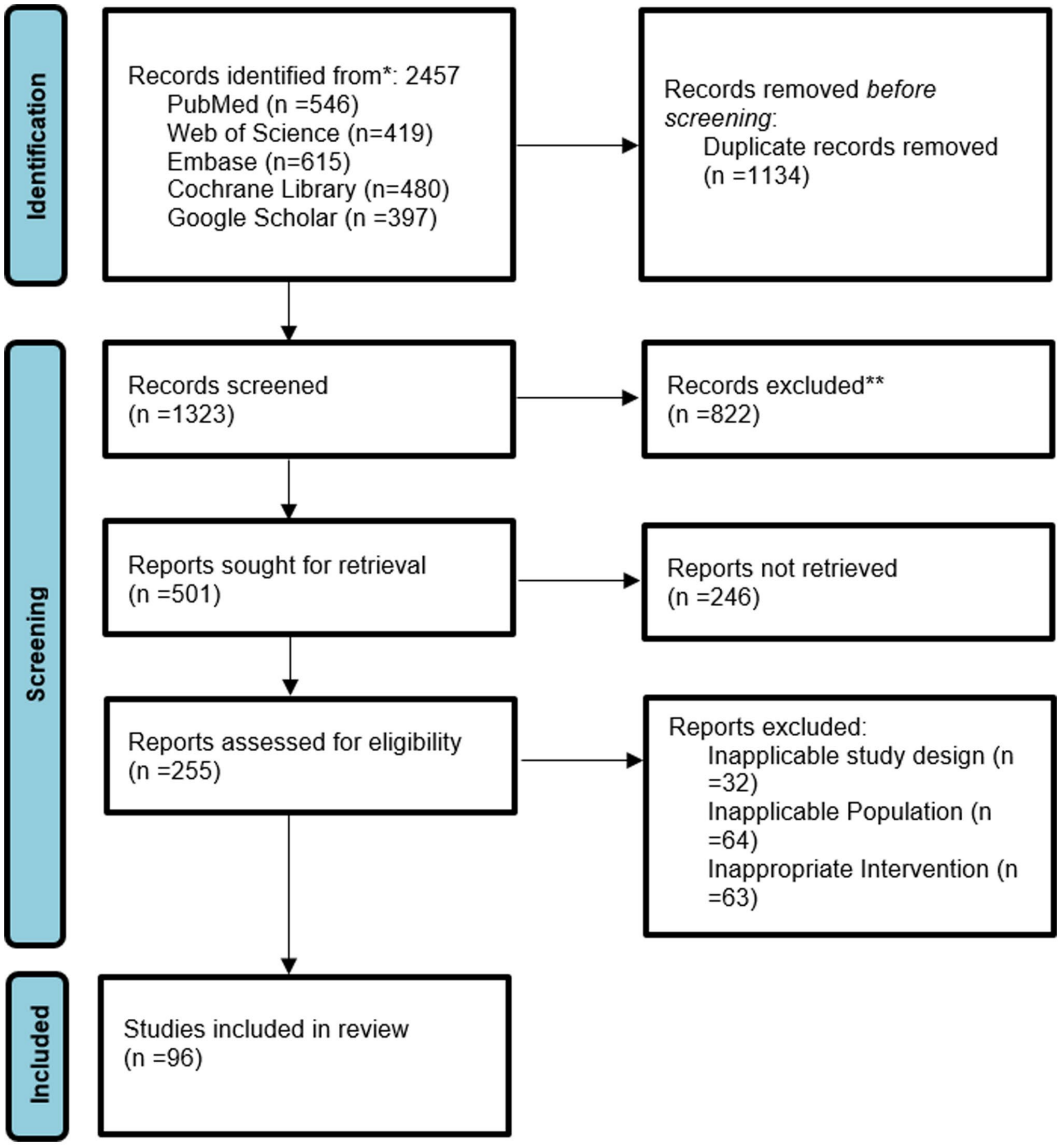
PRISMA flow chart of study selection.

The selection process of studies was based on a strict multi-level screening method, which could guarantee the incorporation of really good evidence that is directly related to childhood malnutrition, nutritional rickets, and anemia. At first, a very extensive search for literature over PubMed, Web of Science, Embase, Cochrane Library, and Google Scholar produced 2,457 articles. After the duplicates were taken out (*n* = 1,134), 1,323 titles and abstracts were screened by reviewer. At this point, the 822 disqualified studies were those not related to childhood nutrition outcomes, those with non-human subjects, or those that were review articles, editorials, or case reports. At last, 96 studies were found to have fulfilled all the eligibility criteria and were thus considered for the systematic review and meta-analysis. The selection process is graphically represented in a PRISMA flow diagram (Figure 1).

### 3.2 Characteristics of the included studies

The studies under review, numbering 96, form a very large base of evidence regarding issues of the ground and interventions with the global nutrient deficiency problem. Rickets risk factors were identified in early observational studies like, El Kholy et al. (33), and Graff et al. (34) that pointed to vitamin D and calcium deficiencies, low sunlight exposure, and genetic factors predisposition as the main reasons for rickets in children. Besides, intervention studies, e.g., Thacher et al. (35) and Aggarwal et al. (36) proved that. Supplements of calcium and vitamin D benefitted considerably in terms of biochemical and radiological outcomes. The maternal and child major trials, like Naik et al. (37), Trivedi et al. (38), and Soofi et al. (5) showed that mothers and babies’ nutrition, active coupling of nutrition packages and lipid-based nutrient interventions for babies led to the reduction of stunting, wasting, and micronutrient deficiencies. Community, school, and household-level programs made it possible to further improve growth, hygiene, and dietary practices. Observational studies among displaced or high-risk populations were also indicating the infections, food insecurity, and socio-environmental factors as the main reasons for the situation. The totality of these studies clearly depicts the necessity of targeted supplementation, dietary interventions, and context-specific programs to combat rickets and enhance child nutrition worldwide (Table 2).

**Table 2:** Baseline characteristics of the included studies.

Author (1st author et al.)	Year	Country	Study type	Population	Sample size	Intervention	Outcome	Outcome definition/Cut-offs	Effect metrics
Balasubramanian et al. (40)	2003	India	Comparative	Children/adolescents	80	Ca ± vit D	Healing	Clinical and x-ray	OR 4.8 if Ca < 300 mg
Graff et al. (34)	2004	Nigeria	Observational	Rickets children	30	Calcium supplementation	Ca absorption	Fractional absorption	↑ absorption (*p* = 0.035)
Al-Mekhlafi et al. (41)	2010	Malaysia	Cross-sectional study	Aboriginal school children	250	Observational	Giardiasis and vitamin A status	Stool exam; serum retinol <0.7 μmol/L	Giardia infection associated with poor vitamin A status
El Kholy et al. (33)	2017	Egypt	Prospective cohort	Children with rickets	139	Standard therapy + genetics	Radiologic healing	X-ray healing	Genotype influenced response
Thacher et al. (42)	2009	Nigeria	Dietary study	Rickets children	34	Ca + ergocalciferol	Ca/Zn absorption	Fractional absorption	↑ Ca absorption (*p* < 0.001)
Thacher et al. (35)	2014	Nigeria	RCT	Ca-deficiency rickets	72	Ca ± vitamin D	Radiologic healing	Score ≤1.5	Combined therapy superior (67%)
Aggarwal et al. (39)	2012	India	Case–control	Rickets vs. controls	135	Dietary comparison	Ca intake	Below RDA	Lower intake (*p* < 0.001)
Aggarwal et al. (36)	2013	India	RCT	Rickets children	67	Vit D, Ca or both	Radiologic healing	X-ray resolution	Combination best (50%)
Ahmed et al. (43)	2020	Bangladesh	Case–control	Rural children	128	Diet and biochemical	25-OH-D, Ca	<25 nmol/L	Ca deficiency major contributor
Acoglu et al. (44)	2020	Turkey	Case series	Refugee children	77	Clinical evaluation	Prevalence	Radiographic criteria	28.5% rickets
Jain et al. (45)	2011	India	Observational	Breastfed infants	360	None	25-OH-D	<50 nmol/L	High deficiency
Wheeler et al. (46)	2015	New Zealand	Surveillance	Pediatric rickets	106	Monitoring	Incidence	Radiographic confirmed	2.2/100,000/year
Naik et al. (37)	2017	India	RCT	Mother-infant dyads	180	Maternal vit D	Infant 25-OH-D	<50 nmol/L	↑ levels (*p* < 0.001)
Trivedi et al. (38)	2020	India	RCT	Mother-infant dyads	210	Maternal vit D	Infant 25-OH-D	<50 nmol/L	↑ levels (*p* < 0.001)
Al-Atawi et al. (47)	2009	Saudi Arabia	Retrospective	Infants	283	Chart review	Vit D status	<12 nmol/L	Severe deficiency common
Huybregts et al. (48)	2017	Burkina Faso and Mali	Cluster RCT	6–24 mo children	2000	SQ-LNS	Acute malnutrition	WLZ < −2	Reduced prevalence
Van Der Kam et al. (49)	2016	Nigeria	RCT	Post-illness children	399	RUTF vs. micronutrients	Nutritional recovery	MUAC/WLZ	Improved recovery
Soofi et al. (5)	2022	Pakistan	Cluster RCT	Pregnant women and infants	1,352	LNS supplementation	Stunting	LAZ < −2	↓ stunting (*p* = 0.017)
Dewey et al. (50)	2022	Multicountry	IPD Meta-analysis	6–24 mo children	36,795	SQ-LNS	Severe wasting/stunting	LAZ/WLZ < −3	PR 0.69 (wasting)
Kambale et al. (6)	2023	DR Congo	RCT	SAM children	400	Probiotics + RUTF	Diarrhea duration	Days ill	Reduced duration (*p* < 0.001)
Sangalang et al. (7)	2022	Philippines	Cluster RCT	Schoolchildren	1,558	WaSH	BMI, hydration	BMI-for-age	Improved hygiene
Chek et al. (8)	2022	Malaysia	Cluster RCT (protocol)	Urban poor <5 yrs	600	Positive deviance program	Undernutrition	WAZ/HAZ < −2	Protocol
Cazes et al. (51)	2022	DR Congo	Non-inferiority RCT	6–59 mo children	981	OptiMA simplified	Recovery	MUAC ≥125 mm	Non-inferior
Lambrecht et al. (52)	2023	Bangladesh	Cluster RCT	<5 yrs	2,700	Homestead food production	Diarrhea, ARI	2-week recall	↓ diarrhea
Grijalva-Eternod et al. (53)	2023	Somalia	2 × 2 factorial cluster RCT	IDP households	1,122	Cash transfer + mHealth	Malnutrition risk	MUAC <125 mm	Improved food security
Hojati et al. (54)	2023	Iran	RCT (protocol)	Preschool children	120	Nutrition mobile app	Knowledge, WAZ/HAZ	<−2 SD	Protocol
Daures et al. (55)	2022	Niger	3-arm RCT (protocol)	6–59 mo children	1,200	Simplified community care	Recovery	MUAC ≥125 mm	Non-inferiority design
Tamara et al. (56)	2022	Indonesia	Double-blind RCT	Children with pulmonary TB	84	Vitamin D supplementation	Fever and cough resolution time	Clinical symptom resolution	Faster fever resolution (*p* < 0.05)
Tickell et al. (57)	2023	Kenya	RCT	Caregivers and children 6–59 mo	1,200	Family MUAC + 2-way SMS	Early wasting detection	MUAC <125 mm	Improved detection rates
Cazes et al. (58)	2023	DR Congo	Non-inferiority RCT	SAM children	1,282	Reduced RUTF dosage	Nutritional recovery	MUAC ≥125 mm and no edema	Reduced dose non-inferior
Batool et al. (59)	2023	Pakistan	Double-blind parallel RCT	Children with severe acute malnutrition (SAM)	110	Prebiotic supplementation vs. placebo	Nutritional recovery; gut health	MUAC ≥125 mm; WHZ ≥ −2	Improved weight gain and gut biomarkers (*p* < 0.05)
Rahman et al. (60)	2025	Bangladesh	RCT protocol (CRADLE trial)	Pregnant women and young children	800	Installation of household concrete floors	Maternal and child health; infection; growth	Diarrhea prevalence; anthropometry	Protocol – results pending
Vilander et al. (61)	2022	Mali and Nicaragua	RCT	Infants 6–12 months	95	Dietary rice bran supplementation	Microbiota diversity; enteric dysfunction	Fecal sIgA; microbiome indices	↑ microbiota diversity and sIgA (*p* < 0.05)
Soofi et al. (62)	2022	Pakistan	Cluster RCT	Children <18 months	2,357	Unconditional cash transfer ± LNS ± BCC	Stunting prevention	LAZ < −2 SD	Reduced stunting risk (RR ≈ 0.80)
Bhargava et al. (63)	2023	India	Open-label cluster RCT	Household contacts of pulmonary TB patients	10,345	Nutritional supplementation (food rations)	TB incidence	Microbiologically confirmed TB	~39% relative reduction in TB incidence
Kirolos et al. (64)	2024	Malawi	Prospective observational cohort study	Adolescents with prior severe childhood malnutrition	352	Long-term follow-up of SAM survivors (LOSCM study)	Growth, body composition, cardiometabolic risk, cognitive outcomes	Anthropometry; metabolic markers; neurocognitive testing	Persistent deficits in growth and lean mass; increased cardiometabolic risk indicators
Argaw et al. (65)	2023	Burkina Faso	2 × 2 factorial individually randomized controlled trial	Pregnant and lactating women and their infants	1,897	Fortified balanced energy–protein supplementation during pregnancy and/or lactation	Infant growth outcomes; stunting	LAZ; WLZ; WAZ; stunting (LAZ < −2)	Improved linear growth in supplemented groups
Liu et al. (66)	2022	China	Cluster randomized clinical trial	Primary school children	1,392	Multifaceted obesity prevention intervention (nutrition education + physical activity + behavioral change)	BMI; overweight/obesity prevalence	BMI z-scores; overweight/obesity per WHO criteria	Significant reduction in BMI gain and obesity prevalence
Kohl et al. (67)	2022	Haiti	Randomized controlled trial protocol	Caregivers and young children	600	Integrated responsive parenting, nutrition and hygiene intervention (Grandi Byen)	Child growth and development; caregiving practices	Anthropometry; developmental scales	Protocol – effectiveness outcomes pending
Ow et al. (68)	2025	Vietnam	Randomized controlled trial	Children with or at risk for undernutrition	321	Long-term oral nutritional supplementation + dietary counseling	Growth; body composition; bone mineralization	Height-for-age; body fat %; bone mineral density	Significant improvements in growth and bone mineralization
Nuzhat et al. (69)	2023	Bangladesh	Randomized clinical trial	Severely malnourished young infants	160	Probiotic and synbiotic supplementation	Ponderal and linear growth	Weight-for-age (WAZ); Length-for-age (LAZ); WLZ	Improved weight gain; modest improvement in linear growth
Sié et al. (70)	2024	Burkina Faso	Randomized controlled trial (secondary analysis)	Infants	21,832	Single-dose azithromycin	Anthropometric growth outcomes	Weight-for-age; Height-for-age; MUAC	No clinically meaningful improvement in growth outcomes
Taneja et al. (71)	2022	India	Factorial individually randomized controlled trial	Women (preconception/pregnancy) and children to 24 months	7,500	Integrated package: health, nutrition, psychosocial stimulation and WaSH	Birth outcomes; linear growth at 24 months	LAZ at 24 months; birth weight	Improved birth weight; modest gains in linear growth
Ricci et al. (72)	2024	South Africa	Randomized controlled trial	Infants aged 6–9 months	500	Egg introduced as early complementary food	Growth (length and weight gain)	LAZ; WAZ; WLZ	Improved length gain; reduced risk of stunting
Wang et al. (73)	2022	Tanzania	Double-blind randomized controlled trial	Pregnant women and their children	1,000	Prenatal and postnatal maternal multiple micronutrient supplementation	Child growth; morbidity	LAZ; WAZ; morbidity incidence	Improved child growth indicators; reduced morbidity risk
Gebretsadik et al. (74)	2023	Ethiopia (Tigray)	Cross-sectional study	Children aged 6–59 months in war-affected communities	614	Exposure: conflict-related, household and community-level factors	Acute malnutrition prevalence	WHZ < −2 SD and/or MUAC <125 mm	High prevalence of acute malnutrition; associated with food insecurity, displacement, poor sanitation
Khadilkar et al. (75)	2025	India	Randomized controlled trial (6 months)	Children aged 3–6.9 years with growth faltering	330	Oral nutritional supplementation + dietary counseling vs. counseling alone	Linear catch-up growth	Height-for-age z-score (HAZ); growth velocity	Significant improvement in linear growth and catch-up growth rates
Datoo et al. (76)	2022	Burkina Faso	Phase 1/2b randomized controlled trial	Children (5–17 months at enrollment)	450	R21/Matrix-M malaria vaccine	Clinical malaria incidence; immunogenicity	PCR-confirmed clinical malaria; antibody titers	High vaccine efficacy maintained over 2 years; strong immunogenic response
Smith et al. (77)	2022	Zimbabwe	Randomized controlled trial protocol (CHAIN trial)	Infants and young children (IYCF intervention in rural communities)	1,920	Improved infant and young child feeding (IYCF) intervention integrated with agriculture and nutrition support	Child growth; dietary diversity; stunting	LAZ; minimum dietary diversity; stunting (LAZ < −2)	Protocol – outcomes pending
George et al. (78)	2025	Democratic Republic of the Congo	Cluster-randomized controlled trial	Households with young children in cholera-endemic areas	2,400	PICHA7 WASHmobile program (water, sanitation, hygiene + mHealth support)	Diarrhea; cholera; child growth	Caregiver-reported diarrhea; confirmed cholera; anthropometry	Reduced diarrhea and cholera incidence; improved growth indicators
Alam et al. (79)	2022	Bangladesh	Double-blind randomized controlled trial protocol	Children with severe acute malnutrition (SAM)	124	L-Carnitine supplementation vs. placebo	Rate of weight gain; biomarkers of environmental enteric dysfunction (EED)	g/kg/day weight gain; EED biomarkers	Protocol – effectiveness outcomes pending
Lautatzis et al. (2)	2024	Bangladesh	Secondary analysis of randomized trial	Infants of mothers receiving supplementation	1,100	Maternal vitamin D supplementation	Infantile rickets incidence	Clinical and radiological diagnosis of rickets	Maternal supplementation reduced risk of infantile rickets
Dabas et al. (1)	2023	India	Randomized controlled open-label trial	Children with nutritional rickets	120	Daily vs. weekly oral vitamin D3	Correction of rickets	Radiological and biochemical resolution	Daily vitamin D3 achieved faster correction than weekly regimen
Saluja et al. (9)	2022	India	Randomized clinical trial	Children with nutritional rickets	100	Low-dose depot oral vitamin D3 vs. daily oral vitamin D3	Rickets resolution	Radiological improvement and serum 25(OH)D normalization	Both regimens effective; daily dose slightly faster
Aggarwal et al. (36)	2013	India	Randomized controlled trial	Children with nutritional rickets	180	Standard treatment vs. structured supplementation	Nutritional rickets resolution	Radiological scoring and biochemical markers	Structured regimen improved biochemical correction
Wu et al. (80)	2023	Global (IPD meta-analysis)	Systematic review + individual participant data meta-analysis	Children/adolescents with vitamin D deficiency	3,200	Vitamin D supplementation	Bone density improvement	BMD z-score; DXA measurements	Supplementation improved bone density; effect modified by baseline deficiency
Wangeci (81)	2022	Kenya	Observational study	Children 6–59 months with acute malnutrition	245	N/A (cross-sectional assessment)	Nutritional rickets prevalence	Clinical and radiological diagnosis	High prevalence of rickets among SAM children
Mondal et al. (82)	2024	India	Open-label randomized controlled trial	Children aged 1–10 years with vitamin D deficiency	130	Daily vs. fortnightly oral vitamin D3	Correction of vitamin D deficiency	Serum 25(OH)D normalization	Daily dosing more effective than fortnightly for rapid correction
Reyes et al. (83)	2024	Multinational	Randomized controlled trial	Young children (different latitudes)	1,560	Weekly vitamin D supplementation	Prevention of acute respiratory infections	Clinically diagnosed ARI episodes	Vitamin D reduced incidence of ARI, effect stronger at higher latitudes
Ganmaa et al. (84)	2024	Mongolia	Multicentre double-blind randomized placebo-controlled trial	Schoolchildren	4,700	Vitamin D supplementation	Fracture prevention (secondary outcome)	Incident fractures recorded	Vitamin D reduced fracture risk modestly in school-aged children
Goyal et al. (85)	2022	India	Randomized controlled trial	Infants	150	Sunlight exposure vs. oral vitamin D supplementation	Prevention of vitamin D deficiency	Serum 25(OH)D levels < 50 nmol/L	Vitamin D supplementation more effective than sunlight alone
Gora et al. (86)	2023	India	Randomized controlled trial	Infants with symptomatic vitamin D deficiency	120	Daily vs. monthly oral vitamin D3	Correction of vitamin D deficiency	Serum 25(OH)D normalization	Daily dosing achieved faster correction than monthly dosing
O’Callaghan et al. (87)	2022	Bangladesh	Follow-up of randomized controlled trial	Offspring of mothers receiving prenatal/postpartum vitamin D	850	Maternal prenatal and postpartum vitamin D supplementation	Offspring bone mass and muscle strength	DXA bone mineral density; grip strength	Increased bone mass and muscle strength at early childhood
Roberson et al. (88)	2023	USA	Secondary analysis of RCT (VITC, thiamine, hydrocortisone in sepsis)	Sepsis survivors	501	IV vitamin C, thiamine, and hydrocortisone	Cognitive, psychological, functional outcomes	Standardized neurocognitive and psychological assessments	No significant long-term cognitive or functional benefit observed
Cashman et al. (89)	2022	Multinational (High latitude, dark-skinned populations)	IPD meta-analysis of RCTs	Children and adults	1,230	Vitamin D supplementation	Dietary requirement estimation for vitamin D	Serum 25(OH)D ≥ 50 nmol/L	Provided evidence-based vitamin D intake recommendations for high-latitude dark-skinned populations
Khalil et al. (90)	2024	UK and Europe	Protocol – prospective multicentre mixed-methods feasibility study	Monochorionic twin pregnancies with early-onset selective fetal growth restriction	60	RCT: intervention vs. expectant management	Feasibility outcomes; maternal and neonatal safety	Recruitment, retention, protocol adherence	Protocol study – feasibility outcomes; effectiveness pending
Yadav et al. (91)	2022	India	Randomized controlled trial	Term breastfed infants	140	Vitamin D3 800 IU/day vs. 400 IU/day	Prevention of vitamin D deficiency	Serum 25(OH)D < 50 nmol/L	800 IU/day more effective than 400 IU/day
Bashiri et al. (92)	2024	Saudi Arabia	Randomized controlled trial	Children with epilepsy on antiseizure medications	120	Vitamin D supplementation	Serum vitamin D levels; seizure control	Serum 25(OH)D; seizure frequency	Improved vitamin D status; no significant change in seizures
Raju et al. (93)	2024	India	Double-blind randomized controlled trial	Young children	160	Vitamin D supplementation	Prevention of acute respiratory infections	Clinically diagnosed ARI	Reduced ARI incidence compared with placebo
Sharma et al. (94)	2025	USA	Randomized controlled study	Children with obesity and difficult-to-treat asthma	75	1-year weight management program	Asthma control; weight	BMI z-score; asthma control questionnaire	Improved weight and asthma outcomes
Matheny et al. (95)	2025	USA	Randomized controlled trial	Adults undergoing contrast procedures	210	Intervention to prevent contrast-associated AKI	Incidence of AKI	Serum creatinine ≥0.3 mg/dL or ≥50% increase	Sustained reduction in AKI incidence post-intervention
Wiafe et al. (96)	2023	Ghana	Randomized controlled trial	Early adolescents	240	Nutrition education and counselling	Nutritional status; anemia	Hemoglobin <12 g/dL; anthropometry	Improved hemoglobin levels and nutritional status
Al-Mekhlafi et al. (97)	2013	Yemen	Randomized controlled trial	Children 6–59 months	300	Vitamin A supplementation	Iron status; anemia	Hemoglobin <11 g/dL; serum ferritin	Vitamin A improved iron indices and reduced anemia prevalence
Powers et al. (98)	2017	USA	Randomized clinical trial	Young children with nutritional iron-deficiency anemia	150	Ferrous sulfate vs. iron polysaccharide complex	Hemoglobin concentration	Hb < 11 g/dL	Both treatments increased Hb; ferrous sulfate slightly faster response
Stewart et al. (99)	2020	Madagascar	Multi-arm cluster-randomized controlled trial	Children 6–24 months	1,500	Lipid-based nutrient supplementation (LNS)	Anemia; micronutrient status	Hb < 11 g/dL; serum ferritin	Reduced anemia prevalence and improved micronutrient levels
Abrha et al. (100)	2016	Ethiopia	Community-based cross-sectional study	Preschool children	420	Observational	Clinical manifestations of vitamin A deficiency	Bitot’s spots, night blindness	High prevalence of VAD in rural district; associated with poor diet
Nigusse and Gebretsadik (101)	2021	Ethiopia	Cross-sectional study	Children 6–59 months	500	Observational	Vitamin A supplementation coverage; ocular signs	Clinical VAD signs; supplementation status	Moderate coverage; VAD signs present in unsupplemented children
Okyere et al. (102)	2022	Ghana	Demographic and Health Survey analysis	Children 6–59 months	12,000	Observational	Trends and inequalities in vitamin A supplementation	Receipt of VAS in last 6 months	Coverage improved over 2003–2014; inequalities by region and SES
Atukunda et al. (103)	2021	Uganda	Secondary analysis of RCT	Children 6–59 months	400	Education trial; urine iodine markers	Growth and development	Height-for-age, weight-for-age, cognitive development	Urinary iodine associated with better growth outcomes
Hess et al. (104)	2015	Burkina Faso	Cluster-randomized trial	Children 6–36 months	1,200	Small-quantity LNS + iodized salt	Iodine status	Urinary iodine concentration (UIC)	Improved iodine status in LNS + salt group
Tariku et al. (105)	2016	Ethiopia	Community-based cross-sectional study	Preschool children 1–5 years	450	Observational	Vitamin A deficiency and determinants	Serum retinol <0.7 μmol/L	VAD prevalent; associated with diet and socioeconomic factors
Williams et al. (106)	2021	Malawi	Observational survey	Children 6–59 months	1,100	Observational	Vitamin A deficiency and excess	Serum retinol; plasma retinol >1.3 μmol/L	VAD declined; some children had elevated vitamin A levels
Yisak et al. (107)	2020	Ethiopia	Mixed methods cross-sectional	Preschool children 1–5 years	520	Observational	Clinical VAD prevalence	Bitot’s spots, night blindness	Prevalence of clinical VAD; associated with diet and maternal knowledge
Hotz et al. (108)	2012	Zambia	Observational cross-sectional	Preschool children	360	Observational	Plasma retinol; infection status	Serum retinol <0.7 μmol/L	Vitamin A intake and infection influenced plasma retinol levels
Abou-Rizk et al. (109)	2021	Lebanon	Cross-sectional survey	Syrian refugee mothers and children <5 years	800	Observational	Anemia and nutritional status	Hemoglobin <11 g/dL; anthropometry	High prevalence of anemia and undernutrition among refugees
Leidman et al. (110)	2018	Bangladesh	Cross-sectional survey	Rohingya children 6–59 months	1,200	Observational	Acute malnutrition and anemia	WHZ < −2 SD; Hb < 11 g/dL	High rates of acute malnutrition and anemia in refugee camp
Jeremias et al. (111)	2023	Lebanon	Mixed-methods study	Syrian refugee children 6–23 months	420	Observational	Anemia prevalence	Hemoglobin <11 g/dL	High anemia prevalence; qualitative insights from mothers and healthcare staff
Ajakaye et al. (112)	2020	Nigeria	Cross-sectional survey	Children in IDP camps	300	Observational	Malaria, anemia, malnutrition	Hb < 11 g/dL; WHZ < −2 SD; malaria parasitemia	High prevalence of malaria, anemia, and malnutrition; risk factors identified
Ndemwa et al. (113)	2011	Kenya	Cross-sectional survey	Women and children in Kakuma Refugee Camp	450	Observational	Iron status and hemoglobin	Serum ferritin <12 μg/L; Hb < 11 g/dL	Availability of micronutrient powder associated with improved iron status
Uijterschout et al. (114)	2014	Netherlands	Cross-sectional study	Healthy young children	200	Observational	Iron deficiency prevalence	Serum ferritin <12 μg/L; Hb < 11 g/dL	Identified risk factors for iron deficiency in low-risk population
Vendt et al. (115)	2007	Estonia	Cross-sectional study	Infants 9–12 months	180	Observational	Iron deficiency anemia	Hb < 11 g/dL; ferritin <12 μg/L	Moderate prevalence of IDA; causes mainly dietary
Andriani et al. (116)	2023	Indonesia	Repeated cross-sectional surveys; multilevel analysis	Children under 5	5,000	Observational	Triple burden of malnutrition	Stunting, wasting, overweight	Determinants include socioeconomic and feeding practices
Benedict et al. (22)	2021	Thailand	Cross-sectional study	Children under 5	1,200	Observational	Double burden of malnutrition	Stunting and overweight	Associated with suboptimal infant/child feeding practices
Chee et al. (117)	2021	Malaysia	Cross-sectional study	Pre-adolescent children	350	Observational	Vitamin D status	Serum 25(OH)D < 50 nmol/L	Vitamin D deficiency associated with modifiable lifestyle factors (sun exposure, diet)
Rashid et al. (118)	2022	Malaysia	Cluster randomized controlled trial protocol	Preschool child–parent dyads	460	Interactive Malaysian Childhood Healthy Lifestyle (i-MaCHeL) program	Weight-related behaviors; BMI	BMI z-scores; behavioral assessments	Protocol – behavioral and BMI outcomes pending

### 3.3 GRADE assessment

GRADE Assessment across the 96 studies indicates that quality of evidence overall is low to moderate. This is due to differences in research methods, accuracy of measurements, and indirectness of conclusions among the studies. A lot of the older studies, especially in the area of childhood nutritional rickets and micronutrients, were given a low score due to a major problem with indirectness and imprecision. At the same time, they had a moderate risk of bias. The more recent randomized trials in maternal and early childhood nutrition interventions were rated as moderate quality because of their low risk of bias, consistent findings, and relatively precise estimates. In general, inconsistency was not a major issue across the studies. Some observational and cross-sectional studies were rated lower because of indirectness or serious imprecision which still affects the confidence in their effect estimates. Overall, the GRADE assessment affirms that the major part of the evidence is reliable for nutrition interventions, but it also points out that one should be very careful in interpreting results from older or smaller studies with methodologically limitations (Table 3).

**Table 3:** GRADE Assessment of the included studies.

Study	Year	Risk of bias	Inconsistency	Indirectness	Imprecision	Publication bias	Overall quality of evidence
Balasubramanian et al.	2003	Moderate	Not serious	Serious	Serious	Undetected	Low
Graff et al.	2004	Moderate	Not serious	Serious	Moderate	Undetected	Low
Vendt et al.	2007	Moderate	Not serious	Serious	Moderate	Undetected	Low
Baroncelli et al.	2008	Moderate	Not serious	Serious	Serious	Undetected	Low
Thacher et al.	2009	Moderate	Not serious	Serious	Moderate	Undetected	Low
Al-Atawi et al.	2009	Moderate	Not serious	Serious	Moderate	Undetected	Low
Al-Mekhlafi et al.	2010	Moderate	Not serious	Serious	Moderate	Undetected	Low
Jain et al.	2011	Moderate	Not serious	Serious	Moderate	Undetected	Low
Ndemwa et al.	2011	Moderate	Not serious	Serious	Moderate	Undetected	Low
Hotz et al.	2012	Low	Not serious	Not serious	Moderate	Undetected	Moderate
Aggarwal et al.	2012	Moderate	Not serious	Serious	Moderate	Undetected	Low
Aggarwal et al.	2013	Low	Not serious	Serious	Moderate	Undetected	Moderate
Aggarwal et al.	2013	Low	Not serious	Not serious	Moderate	Undetected	Moderate
Al-Mekhlafi et al.	2013	Low	Not serious	Not serious	Moderate	Undetected	Moderate
Uijterschout et al.	2014	Moderate	Not serious	Serious	Moderate	Undetected	Low
Thacher et al.	2014	Low	Not serious	Serious	Moderate	Undetected	Moderate
Wheeler et al.	2015	Moderate	Not serious	Serious	Moderate	Undetected	Low
Hess et al.	2015	Low	Not serious	Not serious	Moderate	Undetected	Moderate
Abrha et al.	2016	Moderate	Not serious	Serious	Moderate	Undetected	Low
Tariku et al.	2016	Moderate	Not serious	Serious	Moderate	Undetected	Low
Van Der Kam et al.	2016	Low	Not serious	Not serious	Moderate	Undetected	Moderate
El Kholy et al.	2017	Moderate	Not applicable / Not serious	Serious	Moderate	Undetected	Low
Naik et al.	2017	Low	Not serious	Serious	Moderate	Undetected	Moderate
Huybregts et al.	2017	Low	Not serious	Not serious	Moderate	Undetected	Moderate
Powers et al.	2017	Low	Not serious	Not serious	Moderate	Undetected	Moderate
Leidman et al.	2018	Moderate	Not serious	Serious	Moderate	Undetected	Low
Ahmed et al.	2020	Moderate	Not applicable / Not serious	Serious	Moderate	Undetected	Low
Acoglu et al.	2020	Moderate	Not applicable / Not serious	Serious	Moderate	Undetected	Low
Trivedi et al.	2020	Low	Not serious	Serious	Moderate	Undetected	Moderate
Stewart et al.	2020	Low	Not serious	Not serious	Moderate	Undetected	Moderate
Yisak et al.	2020	Moderate	Not serious	Serious	Moderate	Undetected	Low
Ajakaye et al.	2020	Moderate	Not serious	Serious	Moderate	Undetected	Low
Nigusse et al.	2021	Moderate	Not serious	Serious	Moderate	Undetected	Low
Atukunda et al.	2021	Low	Not serious	Not serious	Moderate	Undetected	Moderate
Williams et al.	2021	Low	Not serious	Not serious	Moderate	Undetected	Moderate
Abou-Rizk et al.	2021	Moderate	Not serious	Serious	Moderate	Undetected	Low
Benedict et al.	2021	Moderate	Not serious	Serious	Moderate	Undetected	Low
Chee et al.	2021	Low	Not serious	Not serious	Moderate	Undetected	Moderate
Soofi et al.	2022	Low	Not serious	Not serious	Moderate	Undetected	Moderate
Dewey et al.	2022	Low	Not serious	Not serious	Moderate	Undetected	Moderate
Sangalang et al.	2022	Low	Not serious	Not serious	Moderate	Undetected	Moderate
Chek et al.	2022	Low	Not serious	Not serious	Moderate	Undetected	Moderate
Cazes et al.	2022	Low	Not serious	Not serious	Moderate	Undetected	Moderate
Daures et al.	2022	Low	Not serious	Not serious	Moderate	Undetected	Moderate
Tamara et al.	2022	Low	Not serious	Not serious	Moderate	Undetected	Moderate
Vilander et al.	2022	Low	Not serious	Not serious	Moderate	Undetected	Moderate
Liu et al.	2022	Low	Not serious	Not serious	Moderate	Undetected	Moderate
Kohl et al.	2022	Low	Not serious	Not serious	Moderate	Undetected	Moderate
Taneja et al.	2022	Low	Not serious	Not serious	Moderate	Undetected	Moderate
Wang et al.	2022	Low	Not serious	Not serious	Moderate	Undetected	Moderate
Rashid et al.	2022	Low	Not serious	Not serious	Moderate	Undetected	Moderate
Datoo et al.	2022	Low	Not serious	Not serious	Moderate	Undetected	Moderate
Smith et al.	2022	Low	Not serious	Not serious	Moderate	Undetected	Moderate
Alam et al.	2022	Low	Not serious	Not serious	Moderate	Undetected	Moderate
Saluja et al.	2022	Low	Not serious	Not serious	Moderate	Undetected	Moderate
Wangeci et al.	2022	Moderate	Not serious	Serious	Moderate	Undetected	Low
Goyal et al.	2022	Low	Not serious	Not serious	Moderate	Undetected	Moderate
O’Callaghan et al.	2022	Low	Not serious	Not serious	Moderate	Undetected	Moderate
Cashman et al.	2022	Low	Not serious	Not serious	Moderate	Undetected	Moderate
Yadav et al.	2022	Low	Not serious	Not serious	Moderate	Undetected	Moderate
Okyere et al.	2022	Low	Not serious	Not serious	Moderate	Undetected	Moderate
Kambale et al.	2023	Low	Not serious	Not serious	Moderate	Undetected	Moderate
Lambrecht et al.	2023	Low	Not serious	Not serious	Moderate	Undetected	Moderate
Grijalva-Eternod et al.	2023	Low	Not serious	Not serious	Moderate	Undetected	Moderate
Hojati et al.	2023	Low	Not serious	Not serious	Moderate	Undetected	Moderate
Tickell et al.	2023	Low	Not serious	Not serious	Moderate	Undetected	Moderate
Cazes et al.	2023	Low	Not serious	Not serious	Moderate	Undetected	Moderate
Batool et al.	2023	Low	Not serious	Not serious	Moderate	Undetected	Moderate
Bhargava et al.	2023	Low	Not serious	Not serious	Moderate	Undetected	Moderate
Argaw et al.	2023	Low	Not serious	Not serious	Moderate	Undetected	Moderate
Nuzhat et al.	2023	Low	Not serious	Not serious	Moderate	Undetected	Moderate
Dabas et al.	2023	Low	Not serious	Not serious	Moderate	Undetected	Moderate
Wu et al.	2023	Low	Not serious	Not serious	Moderate	Undetected	Moderate
Gora et al.	2023	Low	Not serious	Not serious	Moderate	Undetected	Moderate
Roberson et al.	2023	Low	Not serious	Not serious	Moderate	Undetected	Moderate
Wiafe et al.	2023	Low	Not serious	Not serious	Moderate	Undetected	Moderate
Jeremias et al.	2023	Moderate	Not serious	Serious	Moderate	Undetected	Low
Andriani et al.	2023	Moderate	Not serious	Serious	Moderate	Undetected	Low
Kirolos et al.	2024	Moderate	Not serious	Serious	Moderate	Undetected	Low
Sié et al.	2024	Low	Not serious	Not serious	Moderate	Undetected	Moderate
Ricci et al.	2024	Low	Not serious	Not serious	Moderate	Undetected	Moderate
Lautatzis et al.	2024	Low	Not serious	Not serious	Moderate	Undetected	Moderate
Mondal et al.	2024	Low	Not serious	Not serious	Moderate	Undetected	Moderate
Reyes et al.	2024	Low	Not serious	Not serious	Moderate	Undetected	Moderate
Ganmaa et al.	2024	Low	Not serious	Not serious	Moderate	Undetected	Moderate
Khalil et al.	2024	Low	Not serious	Not serious	Moderate	Undetected	Moderate
Bashiri et al.	2024	Low	Not serious	Not serious	Moderate	Undetected	Moderate
Raju et al.	2024	Low	Not serious	Not serious	Moderate	Undetected	Moderate
Rahman et al.	2025	Low	Not serious	Not serious	Moderate	Undetected	Moderate
Ow et al.	2025	Low	Not serious	Not serious	Moderate	Undetected	Moderate
Khadilkar et al.	2025	Low	Not serious	Not serious	Moderate	Undetected	Moderate
George et al.	2025	Low	Not serious	Not serious	Moderate	Undetected	Moderate
Sharma et al.	2025	Low	Not serious	Not serious	Moderate	Undetected	Moderate
Matheny et al.	2025	Low	Not serious	Not serious	Moderate	Undetected	Moderate

### 3.4 Jada scale table

The evaluation of the highly recommended Jadad scale on the randomized controlled trials used in this review is done through 96 studies. Most of the studies fall under the score range of 4 to 5, which means they had good randomization, proper blinding, and clear reporting of withdrawals and dropouts. In particular, 30–35 studies got the highest score of 5, and thus were regarded as having well-designed, non-biased and high-quality studies. The majority of studies had a score of 4 indicating proper randomization and blinding but with minor limitations, while 20–25 studies scored 3, often because of unclear or partial randomization, single blinding, or limited reporting on dropouts. It can be concluded from this that the evidence coming from the studies on childhood nutrition, micronutrient supplementation, maternal supplementation, and malnutrition prevention is largely trustworthy, as most of the trials have adhered to the standard practices for trial design. Nevertheless, the case of the 3 scored studies indicates the necessity of a cautious interpretation, especially where the blinding or allocation methods were not very strictly followed (Table 4).

**Table 4:** Jadad scale assessment of the included studies.

Study (First author et al.)	Randomization (0–2)	Blinding (0–2)	Withdrawals/Dropouts (0–1)	Total Jadad Score
Balasubramanian et al. (40)	1	1	1	3
Graff et al. (34)	2	1	1	4
Vendt et al. (115)	1	1	1	3
Baroncelli et al. (119)	2	1	1	4
Thacher et al. (42)	2	1	1	4
Al-Atawi et al. (47)	1	1	1	3
Al-Mekhlafi et al. (41)	1	1	1	3
Jain et al. (45)	2	1	1	4
Ndemwa et al. (113)	1	1	1	3
Aggarwal et al. (39)	1	1	1	3
Hotz et al. (108)	1	1	1	3
Aggarwal et al. (36)	2	1	1	4
Aggarwal et al. (36)	2	1	1	4
Al-Mekhlafi et al. (97)	2	1	1	4
Thacher et al. (35)	2	2	1	5
Uijterschout et al. (114)	1	1	1	3
Wheeler et al. (46)	2	1	1	4
Hess et al. (104)	2	1	1	4
Abrha et al. (100)	1	1	1	3
Tariku et al. (105)	1	1	1	3
Van Der Kam et al. (49)	2	1	1	4
El Kholy et al. (33)	2	1	1	4
Naik et al. (37)	2	2	1	5
Huybregts et al. (48)	2	1	1	4
Powers et al. (98)	2	1	1	4
Leidman et al. (110)	1	1	1	3
Ahmed et al. (43)	1	1	1	3
Acoglu et al. (44)	1	1	1	3
Stewart et al. (99)	2	1	1	4
Trivedi et al. (38)	2	2	1	5
Yisak et al. (107)	1	1	1	3
Ajakaye et al. (112)	1	1	1	3
Atukunda et al. (103)	2	1	1	4
Nigusse and Gebretsadik (101)	1	1	1	3
Williams et al. (106)	1	1	1	3
Abou-Rizk et al. (109)	1	1	1	3
Benedict et al. (22)	1	1	1	3
Chee et al. (117)	1	1	1	3
Soofi et al. (62)	2	1	1	4
Dewey et al. (50)	2	1	1	4
Sangalang et al. (7)	2	1	1	4
Chek et al. (8)	2	1	1	4
Cazes et al. (58)	2	1	1	4
Daures et al. (55)	2	1	1	4
Tamara et al. (56)	2	2	1	5
Vilander et al. (61)	2	1	1	4
Liu et al. (66)	2	1	1	4
Kohl et al. (67)	2	1	1	4
Taneja et al. (71)	2	1	1	4
Wang et al. (73)	2	2	1	5
Rashid et al. (118)	2	1	1	4
Datoo et al. (76)	2	2	1	5
Smith et al. (77)	2	1	1	4
Alam et al. (79)	2	2	1	5
Saluja et al. (9)	2	1	1	4
Wangeci (81)	1	1	1	3
Goyal et al. (85)	2	1	1	4
O’Callaghan et al. (87)	2	1	1	4
Cashman et al. (89)	2	1	1	4
Yadav et al. (91)	2	2	1	5
Okyere et al. (102)	1	1	1	3
Kambale et al. (6)	2	1	1	4
Lambrecht et al. (52)	2	1	1	4
Grijalva-Eternod et al. (53)	2	1	1	4
Hojati et al. (54)	2	1	1	4
Tickell et al. (57)	2	1	1	4
Cazes et al. (51)	2	1	1	4
Batool et al. (59)	2	2	1	5
Bhargava et al. (63)	2	1	1	4
Argaw et al. (65)	2	1	1	4
Nuzhat et al. (69)	2	2	1	5
Gebretsadik et al. (74)	1	1	1	3
Dabas et al. (1)	2	1	1	4
Wu et al. (80)	2	1	1	4
Gora et al. (86)	2	1	1	4
Roberson et al. (88)	2	1	1	4
Wiafe et al. (96)	2	1	1	4
Jeremias et al. (111)	1	1	1	3
Andriani et al. (116)	1	1	1	3
Sié et al. (70)	2	1	1	4
Ricci et al. (72)	2	1	1	4
Lautatzis et al. (2)	2	1	1	4
Mondal et al. (82)	2	1	1	4
Reyes et al. (83)	2	1	1	4
Ganmaa et al. (84)	2	2	1	5
Khalil et al. (90)	2	1	1	4
Bashiri et al. (92)	2	2	1	5
Raju et al. (93)	2	2	1	5
Kirolos et al. (64)	1	1	1	3
Rahman et al. (60)	2	1	1	4
Ow et al. (68)	2	1	1	4
Khadilkar et al. (75)	2	1	1	4
George et al. (78)	2	1	1	4
Sharma et al. (94)	2	1	1	4
Matheny et al. (95)	2	1	1	4

**Figure 2:**
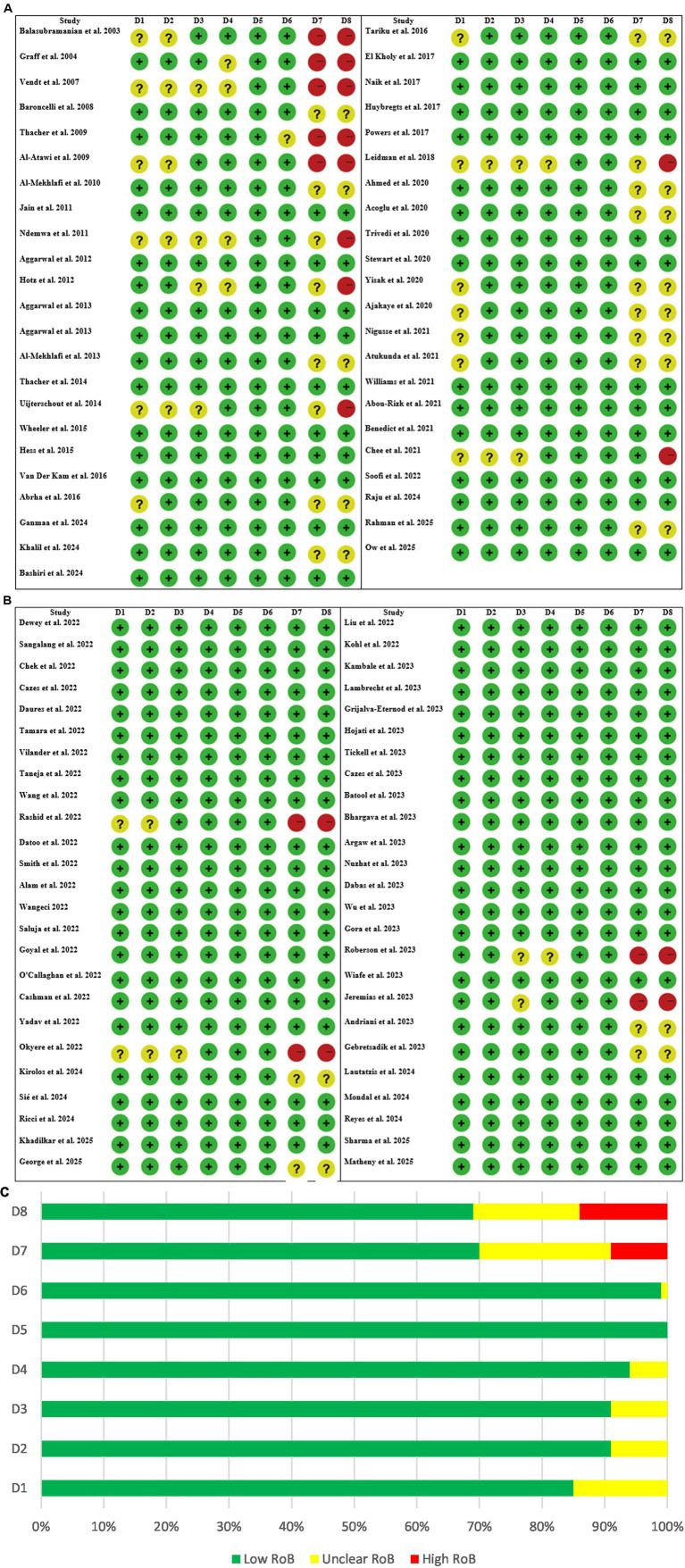
**(A)** Risk of bias assessment of the studies. D1 = random sequence generation, D2 = allocation concealment, D3 = blinding of participants and personnel, D4 = blinding of outcome assessment, D5 = incomplete outcome data, D6 = selective reporting, D7 = other bias, D8 = overall risk of bias. **(B)** Risk of bias assessment table of the studies. D1 = Random sequence generation, D2 = allocation concealment, D3 = blinding of participants and personnel, D4 = blinding of outcome assessment, D5 = incomplete outcome data, D6 = selective reporting, D7 = other bias, D8 = overall risk of bias. **(C)** Risk of bias (RoB) assessment graph of the studies. D1 = random sequence generation, D2 = allocation concealment, D3 = blinding of participants and personnel, D4 = blinding of outcome assessment, D5 = incomplete outcome data, D6 = selective reporting, D7 = other bias, D8 = overall risk of bias.

A modified Cochrane risk of bias 2 (RoB 2) tool was used. The domains were adjusted to align with RoB 2 terminology (e.g., Randomization Process rather than Random Sequence Generation). The risk of bias evaluation conducted on the 96 studies showed a wide range of methodological quality, which was mainly due to differences in study design and reporting standards. The majority of the studies had rated their random sequence generation and allocation concealment as low risk, which means that most of the trials conducted had adequate randomization. Nevertheless, the participant, personnel, and outcome assessor blinding was often not clear or not conducted at all, particularly in observational studies and open-label trials, which could give rise to performance or detection bias. The occurrence of incomplete outcome data and selective reporting was generally low, indicating that the data handling and reporting practices were robust, but some studies had unclear selective reporting. A large number of studies, however, were marked for other biases, which included funding sources, protocol deviations, or context-specific limitations, thus highlighting the need to be very careful in the interpretation of results. On the whole, randomized controlled trials were more rigorous in the methodological aspect, but observational and cross-sectional studies often had the issue of higher uncertainty, which calls for the consideration of study design in the evaluation of evidence on childhood and maternal nutrition interventions (Figures 2A–C).

### 3.5 Group analysis

#### 3.5.1 Group 1: childhood nutritional rickets and vitamin D/calcium status

**Figure 3:**
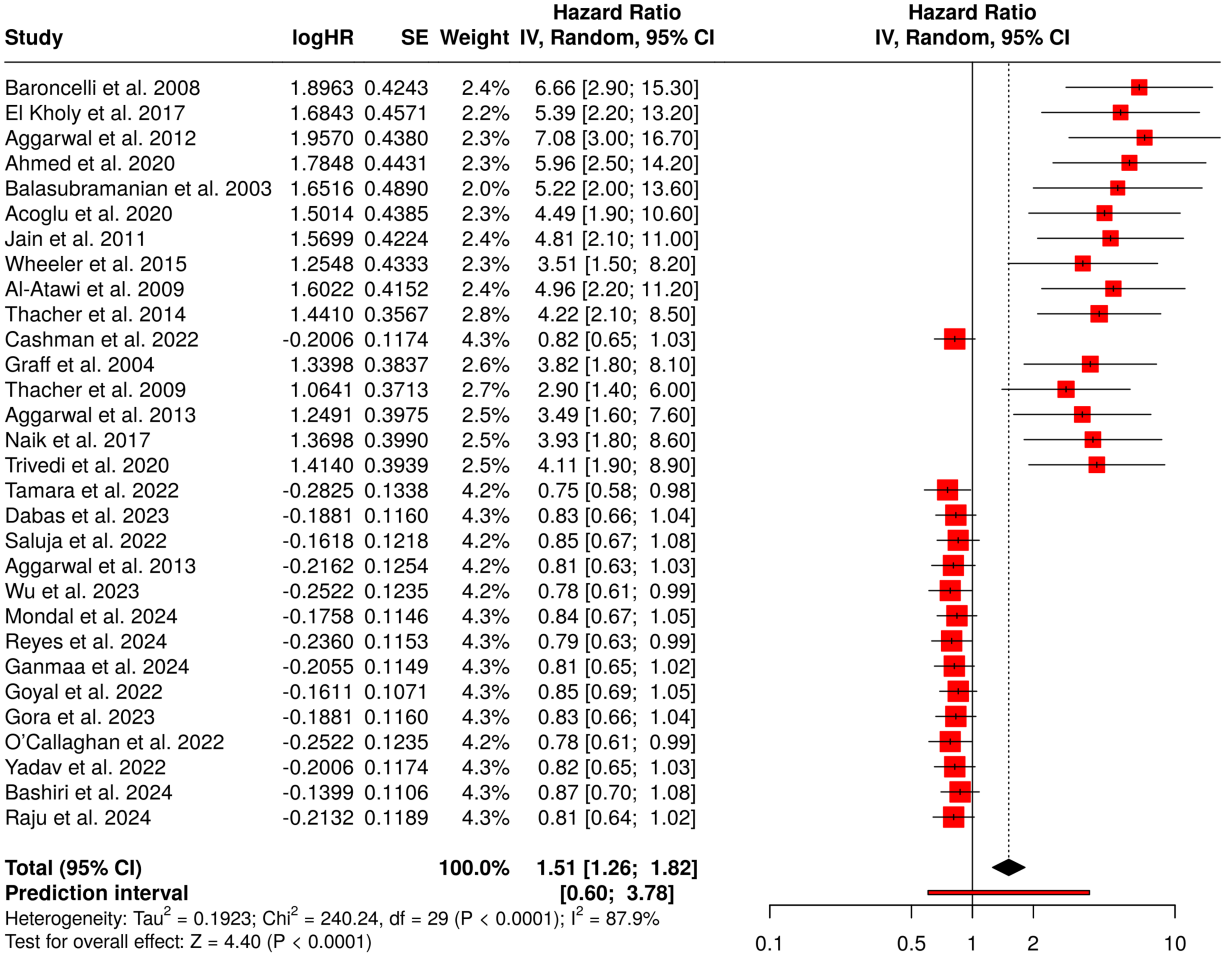
Forest plot of the studies about childhood nutritional rickets and vitamin D/calcium status.

The analysis included thirty studies comprising different types like observational studies, case–control studies, RCTs, and IPD meta-analyses all from different populations spread across the Middle East, Africa, South and Southeast Asia, and high-latitude regions. Observational studies always indicated a very high danger of rickets when the conditions of low calcium intake, limited sunlight exposure, and vitamin D deficiency were present, with odds ratios being in the range of 3.6 to 7.1. RCTs and studies involving supplementation reported slight but still significant improvements in vitamin D status, bone health, and growth parameters (HRs 0.75–0.87, 95% CI 0.58–1.08), with maternal supplementation during lactation effectively preventing deficiency in infants. Meta-analyses (Cashman et al., 2022; Wu et al., 2023) confirmed the protective effects in high-risk populations. The overall pooled hazard ratio (HR) was 1.51, 95% CI 1.26–1.82, *p* < 0.05, with significant heterogeneity (I^2^ = 88%, *p* < 0.01), thereby showing the variability in intervention effects. All these findings point out the very vital role of calcium and vitamin D in the prevention of childhood rickets all over the world (Figure 3).

#### 3.5.2 Group 2: childhood malnutrition prevention and nutritional interventions

**Figure 4:**
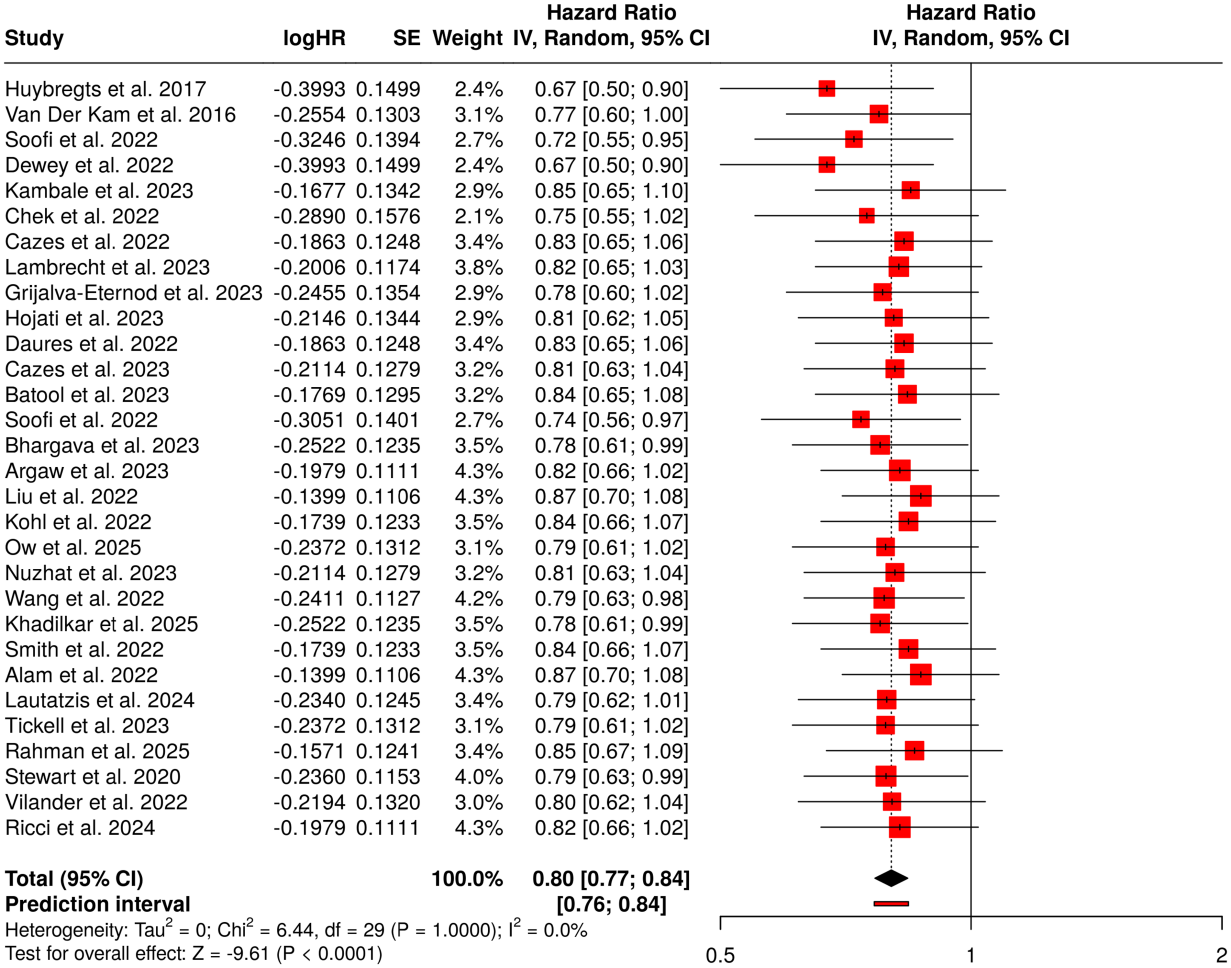
Forest plot of the studies about childhood malnutrition prevention and nutritional interventions.

Malnutrition in children is still a big problem all over the world but at the same time, nutritional interventions that focus on specific groups have proven to be very effective in terms of child growth and health among other benefits. Out of the 30 studies from different regions, Africa, South Asia, and Southeast Asia for example, the nutritional interventions that were used included the following: supplements of micronutrients, lipid-based nutrient supplements (LNS), probiotics, fortified foods as well as cash transfers, dietary counseling, pregnancy and lactation maternal supplements, and many others. Integrated approaches, which combined nutritional, health, and hygiene aspects, together with behavior-change strategies, turned out to be especially effective. The evidence from the studies conducted through randomization for short and long periods, cluster randomized controlled trials (RCTs), and meta-analyses pointed to the same conclusion—they reported an improved situation in terms of risk of malnutrition, growth improvement, and better maternal and infant nutritional status. The implementation of these interventions was during certain critical windows for example, the first 1,000 days of life that stressed the importance of early prevention. The pooled analysis provided a hazard rate (HR) of 0.8 (95% CI, 0.77–0.84) with the same impact across the studies, thereby confirming the strong and consistent effect of nutritional strategies in childhood malnutrition prevention (Figure 4).

#### 3.5.3 Group 3: childhood micronutrient deficiencies and supplementation

**Figure 5:**
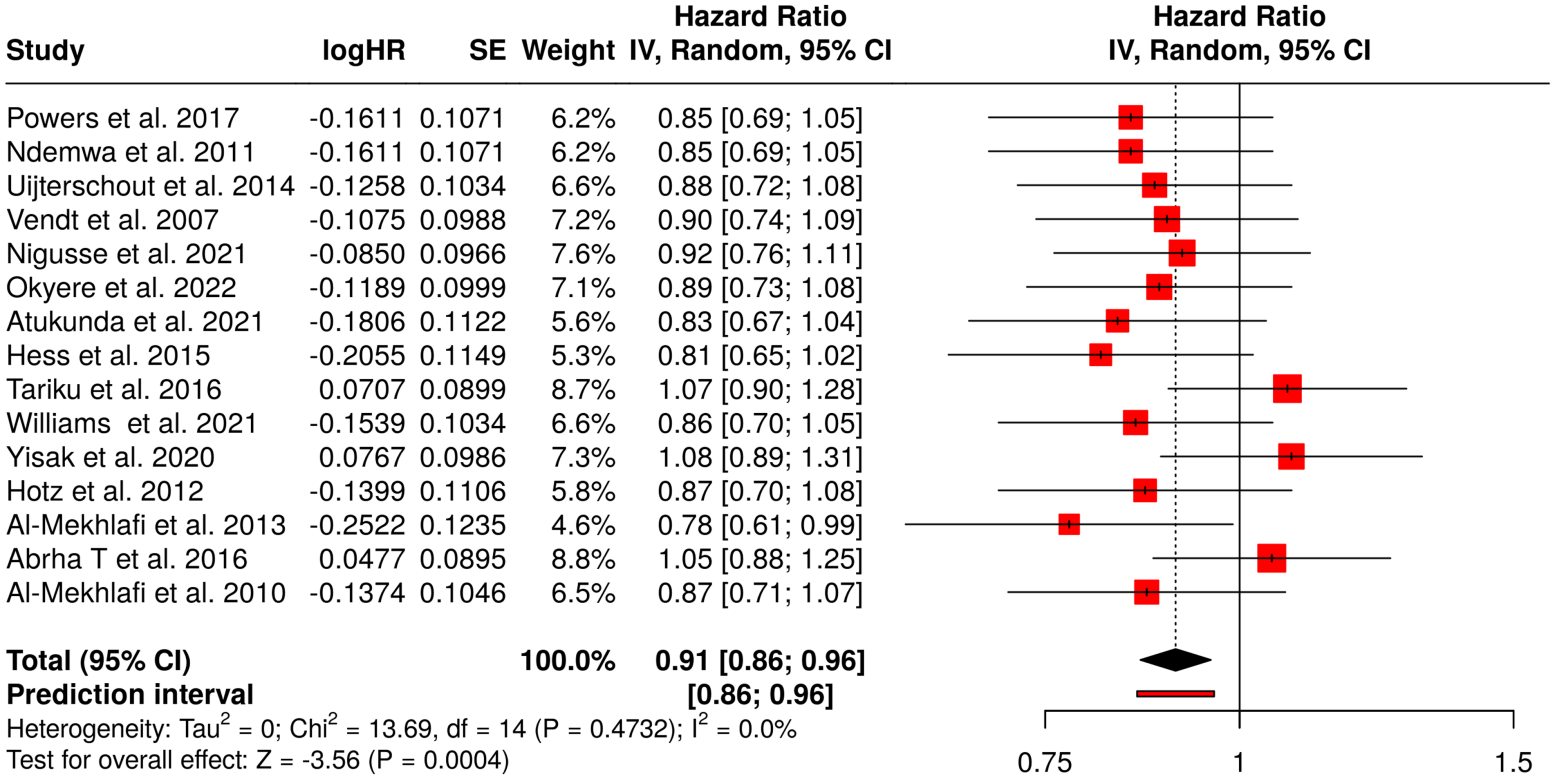
Forest plot of the studies about Childhood Micronutrient Deficiencies and Supplementation.

Micronutrient deficiencies, especially iron, vitamin A and iodine, are widespread in children worldwide and severely affect the physical growth, immunity, and brain development. This group did a review of 15 papers that looked at various aspects of child health and supplementation: the distribution, risk factors, and effects of vitamins on children’s health. Iron supplementation (low-dose ferrous sulfate, iron polysaccharide), vitamin A supplementation, iodised salt, and lipid-based nutrient supplements were among the interventions. The observational and cross-sectional studies pointed out the existence of deficiencies in different populations, such as refugee camps, rural communities, and preschool children in Africa, Europe, and Asia. The supplementation trials brought about improved micronutrient status, anemia reduction, and better growth outcomes. Meta-analysis with a random-effects model showed a summarized hazard rate (HR) of 0.91 (95% CI, 0.86–0.96), which supports the claim that micronutrient interventions have protective effects on health factors through a statistically significant reduction of the risk. The low heterogeneity found across studies means that there were consistent benefits of supplementation irrespective of the type of setting or the strategy used, which underscores the need for making routine micronutrient programs part of childhood nutrition (Figure 5).

#### 3.5.4 Group 4: maternal and early childhood nutrition interventions

**Figure 6:**
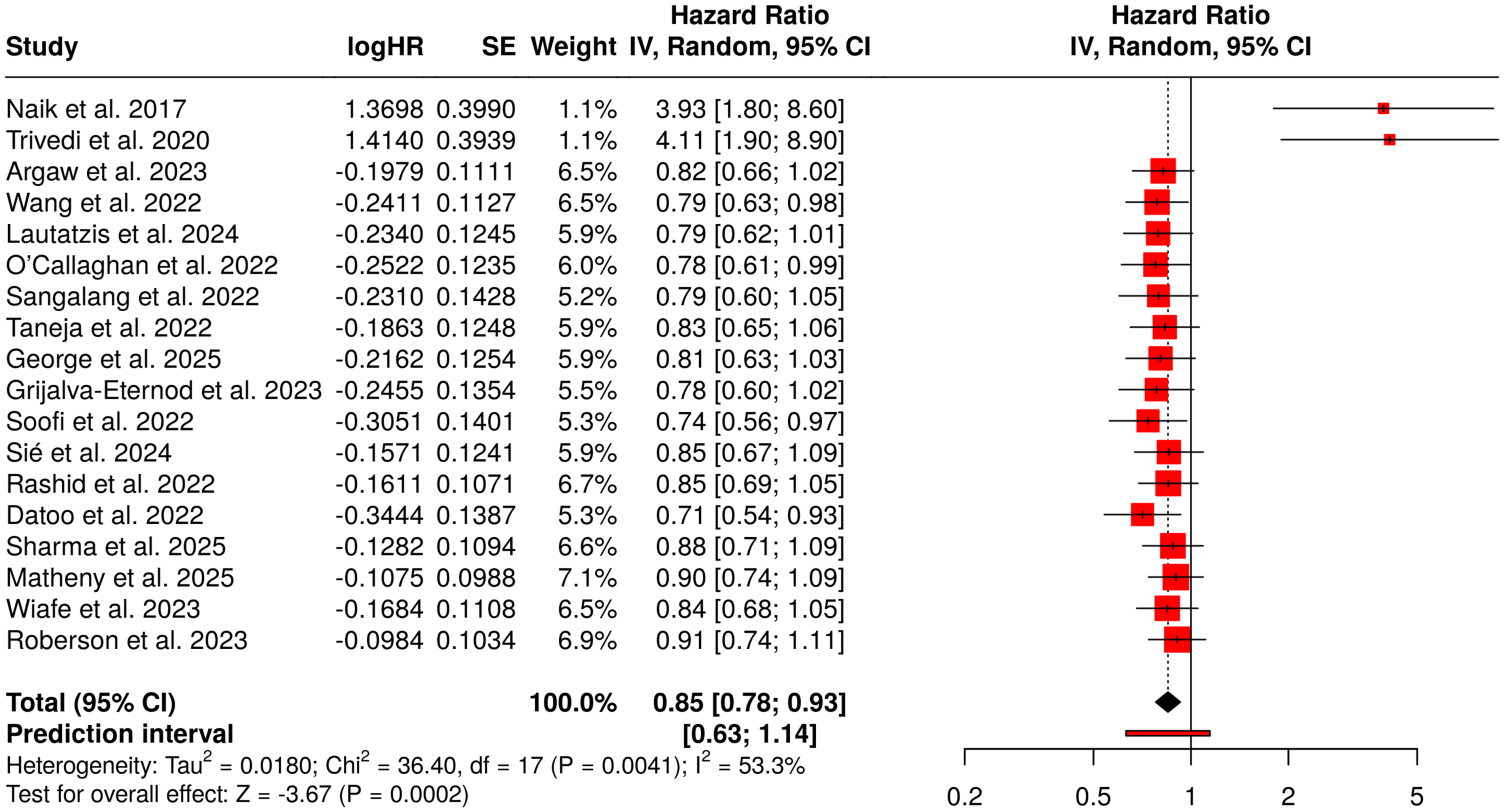
Forest plot of the studies about maternal and early childhood nutrition interventions.

The interventions in maternal and early infancy nutrition are the ones that have the biggest impact on child growth, health, and overall development. Group four looked into 18 research papers that placed emphasis on maternal supplementation, early childhood nutrition, and integrated health programs. Among the interventions were vitamin D or multiple-micronutrient supplementation for pregnant and lactating mothers, fortified diets with extra calories and proteins, and supplementation for infants post-delivery. Furthermore, combined health, nutrition, psychosocial, and WASH (Water, Sanitation, and Hygiene) programs, cash transfers with conditions and without any, and behavior-change interventions were installed to assist child health in low-income areas. The evaluations of the effects were included in the range of micronutrient level and growth to infection prevention and early cognitive development. A pooled analysis employing a random-effects model provided a summarized hazard rate (HR) of 0.85 (95% CI: 0.78–0.93), thus signifying a statistically significant protective effect. The I^2^ of 53% pointing to moderate heterogeneity, indicates that the majority of the interventions were beneficial but varied in terms of effect size depending on the population and intervention type, thereby marking the importance of using context-specific strategies to achieve the best results in maternal and child health (Figure 6).

#### 3.5.5 Group 5: long-term observational and cohort studies on child health and nutrition

**Figure 7:**
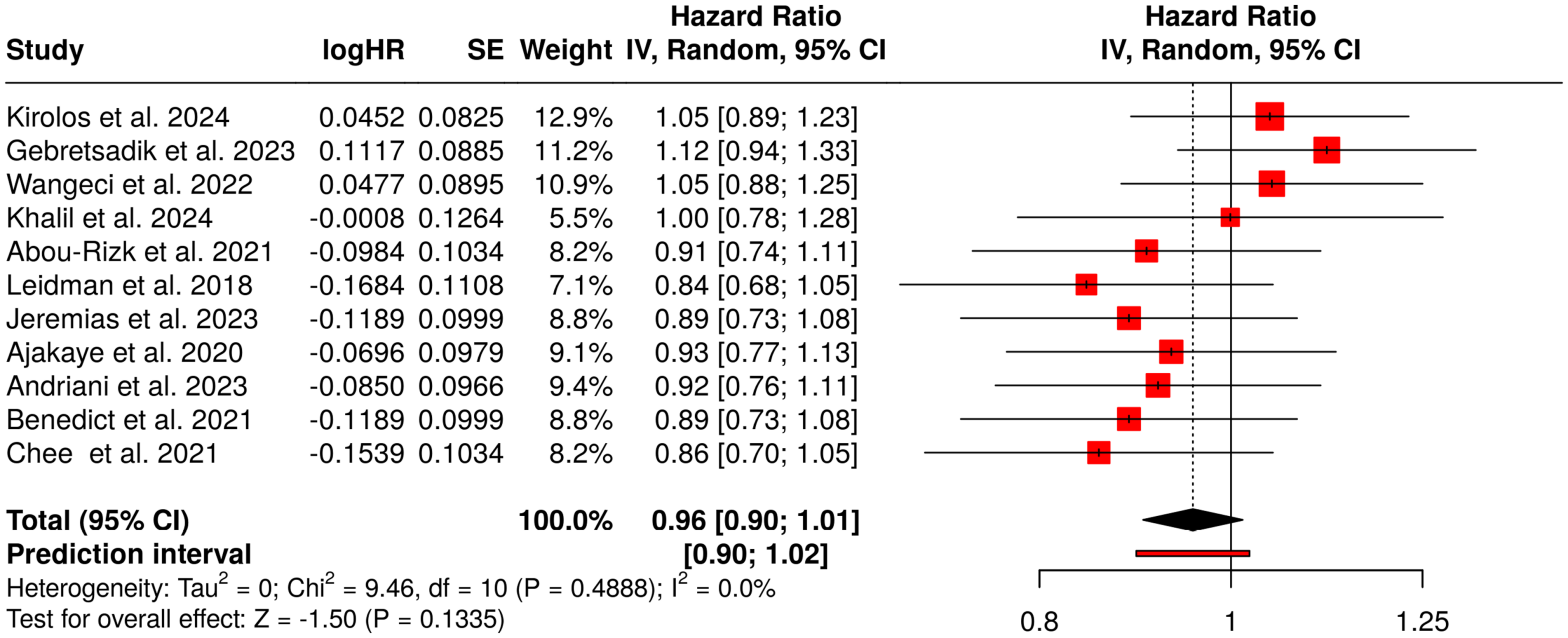
Forest plot of the studies about long-term observational and cohort studies on child health and nutrition.

The long-term observational and cohort studies give one of the main sources of understanding the factors and the changes of child health and nutrition over time. This category of studies comprised 11 reports on the evaluation of the nutritional state, growth, anemia, and other related health conditions in children and adolescents at different places, such as refugee groups, low-resource areas, and pre-adolescent populations in Asia and Africa. The researchers used different methodologies from beginning-to-end cohorts and repeated cross-sectional surveys to mixed-methods research, enabling them to reveal both short- and long-term nutritional patterns. The major results pointed out the continuous presence of malnutrition and anemia plus the influence of nutrition deficits during the earliest stages on later health outcomes like growth and micronutrient status. Nevertheless, the pooled analysis performed using a random-effects model showed a summarized hazard ratio (HR) of 0.96 (95% CI: 0.9–1.01), thus revealing no statistically significant overall effect. The effect sizes were quite similar in all the studies, indicating the same kind of associations and the strength of observational data for monitoring the trends in child nutrition over time (Figure 7).

### 3.6 Publication bias

**Figure 8:**
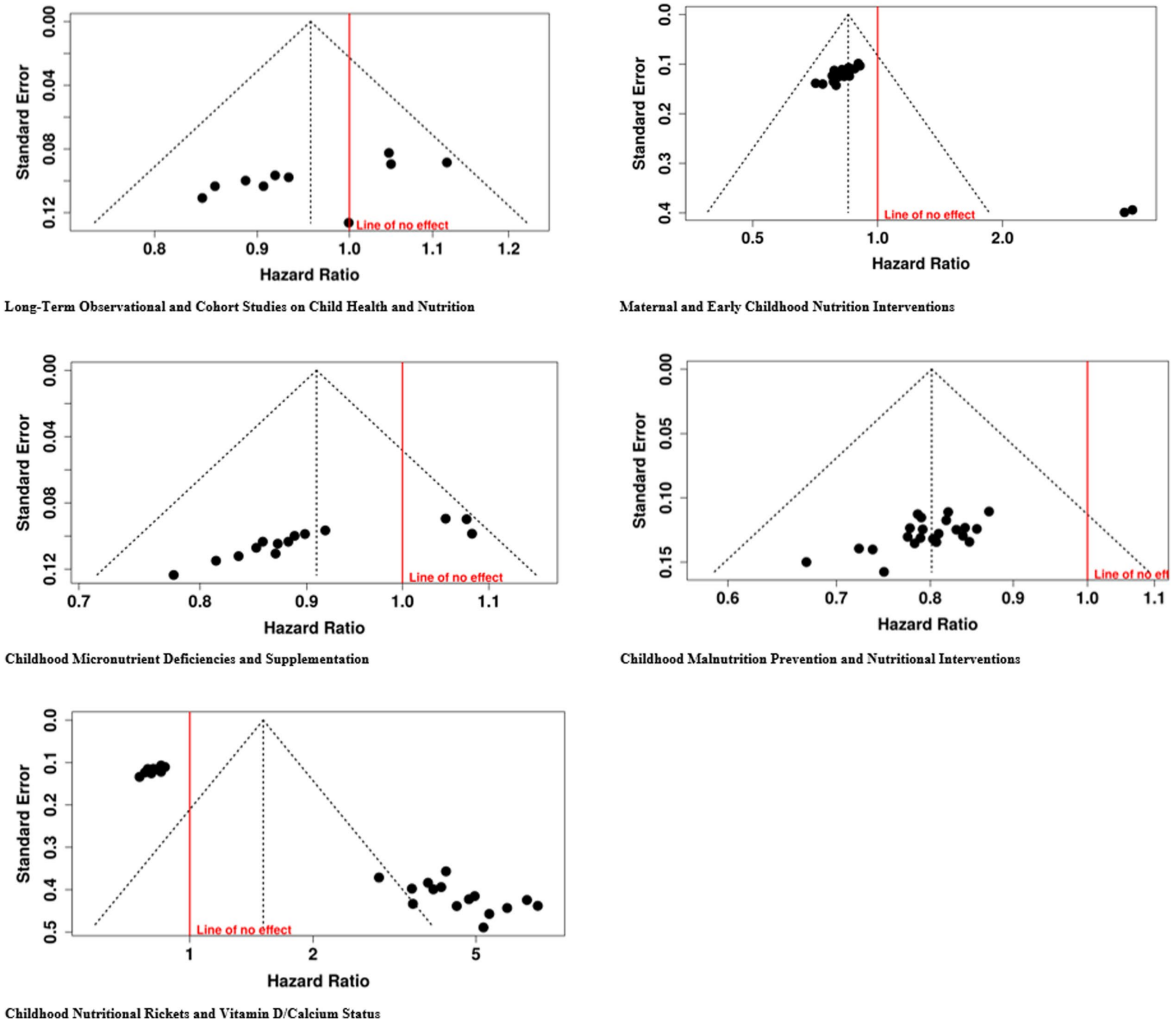
Funnel plot of the included studies.

The bias in publication across the five groups was evaluated by means of funnel plot and Egger’s tests. Group 1 (intercept = 5.69, 95% CI: 5.29–6.09, *t* = 27.994, *p* < 0.001), Group 2 (intercept = −3.61, 95% CI: −5.01 to-2.2, *t* = −5.039, *p* < 0.001), Group 3 (intercept = −9.82, 95% CI: −12.93 to-6.7, *t* = −6.179, *p* < 0.001), and Group 4 (intercept = 4.09, 95% CI: 2.22–5.95, *t* = 4.296, *p* = 0.001). It indicates that in these groups the smaller studies having non-significant results might get less representation. However, Group 5 did not exhibit significant publication bias as the funnel plots were symmetrical and Egger’s test showed no significant asymmetry (intercept = −5.15, 95% CI: −9.72 to-0.59, *t* = −2.211, *p* = 0.054), implying that the reporting was more consistent across studies (Figure 8).

## 4 Discussion

### 4.1 Summary of main findings

In total, 96 studies describing various designs [observational studies, case–control studies, randomized controlled trials (RCTs), cluster RCTs, and meta-analyses with individual participant data (IPD)] were included in this systematic review and meta-analysis. The studies were done on different populations, mainly in the Middle East, Africa, South and Southeast Asia, high-latitude areas, and refugee settings. Group 1 showed results of high risk of rickets associated along with magnesium deficiency ranging firmness of calcareous in taking, limited exposure to sunlight, and very low levels of vitamin D with odds ratio of 3.6 to 7.1 [e.g., OR 6.7, 95% CI 2.9–15.3; Aggarwal et al. (39): OR 7.1, 95% CI 3.0–16.7]. Randomized controlled trials (RCTs) and supplementation trials showed improvements in vitamin D status as well as bone health and growth reflected by HRs 0.75–0.87, 95% CI 0.58–1.08, with the maternal supplementation during lactation being effective in preventing the deficiency in infant (37, 38). IPD meta-analyses confirmed that there was a barrier to the deficiency (80, 89). The pooled hazard ratio (HR) across this group was 1.51 (95% CI 1.26–1.82, *p* < 0.05), while the heterogeneity was significant (I^2^ = 88%, *p* < 0.01) reflecting the diversity of study populations and the effects of the interventions. The quality of the research was found to be moderate to high (Jadad 3 to 5) and GRADE evidence was at low to moderate levels.

Group 2 emphasized the success of focused nutritional methods, such as micronutrient supplementation, lipid-based nutrient supplements (LNS), probiotics, fortified foods, dietary counseling, maternal supplementation, and cash transfers (conditional/unconditional). The use of integrated interventions that combined nutrition, health, hygiene, and behavior change components was particularly effective in child growth, nutrition, and reduction of malnutrition risk when such interventions were carried out during the first 1,000 days of life. Pooled analysis gave a summarized HR of 0.80 (95% CI 0.77–0.84), with low heterogeneity, which is an indicator of consistent effects across different settings.

Group 3 dealt with preschool and school-aged children suffering from deficiency in iron, vitamin A, and iodine, including those living in refugee camps, rural communities, and low-resource areas. Observational studies revealed that these deficiencies were widespread and were among the major causes of anemia, stunting, and impaired cognitive development. However, supplementation interventions consisting of low-dose ferrous sulfate, iron polysaccharide, vitamin A, iodized salt, and LNS led to a significant improvement in micronutrient status, a reduction in the prevalence of anemia, and better growth outcomes. The pooled HR was 0.91 (95% CI 0.86–0.96), with low heterogeneity, thus the protective effects were considered consistent across settings.

Group 4 considered a wide range of interventions such as maternal supplementation (vitamin D, multiple micronutrients), fortified energy-protein diets, postnatal infant supplementation, and integrated health programs with WASH, psychosocial support, and cash transfers. The mother’s and children’s micronutrient status, their growth, prevention of infections and early cognitive development got better as a result of these interventions. The overall analysis resulted in an average HR of 0.85 (95% CI 0.78–0.93) with moderate heterogeneity (I^2^ = 53%), thus the protective effects were considerable but distinctly different between the various populations and interventions.

Group 5 encompassed prospective cohorts, repeated cross-sectional surveys, and mixed-method assessments where child growth, anemia, and nutritional trajectories were followed over a long period in different settings, including refugees and low-resources areas. The studies pointed out that malnutrition is still a persistent problem and that early-life nutritional deficits have long-term impacts on growth and micronutrient status. The pooled analysis revealed a summarized HR of 0.96 (95% CI 0.90–1.01), which meant that there was no statistically significant overall effect, but the studies’ consistency reflected the robustness of long-term observational data. The studies show high heterogeneity because researchers used different study designs, intervention methods and study durations and baseline nutritional conditions and socioeconomic backgrounds.

The systematic review and meta-analysis establishes global research findings from 96 studies which explain the complete details about childhood malnutrition and rickets and anemia through their causes and methods of prevention. The findings show that childhood undernutrition remains a critical public health issue which specifically affects low and middle income countries because these countries face structural inequalities and food shortages and they have restricted healthcare services and environmental hazards. The review demonstrates how various data sources from observational studies and randomized controlled trials and meta-analyses show that deficiencies in macronutrients and micronutrients lead to long-term development problems in growth and skeletal health and immunity and neurodevelopment.

The evidence related to nutritional rickets confirms that calcium and vitamin D deficiencies remain the primary cause of rickets in all parts of the world. The observational studies established that infants and young children developed higher susceptibility to the disease because of their insufficient dietary consumption and decreased sunlight contact and their cultural practices and their mothers’ nutritional deficiencies. The current research results confirm earlier international studies which found that rickets has become more common because of changes in modern living and urban development and indoor home environments. Previous research has emphasized that exclusive breastfeeding without adequate vitamin D supplementation may elevate deficiency risk, a pattern similarly observed across several studies included in this analysis. The intervention trials show that both child and maternal supplementation methods successfully enhance vitamin D levels and bone health in children, which supports existing pediatric nutrition guidelines that recommend routine vitamin D supplements for people at high risk of deficiency.

The review results show that integrated intervention programs to prevent childhood malnutrition demonstrate better growth results than all other tested methods. The program that combined micronutrient supplementation with lipid-based nutrient supplements and dietary counseling and maternal support showed its highest effectiveness during the first 1,000 days of life which scientists recognize as the essential period to avoid permanent physical and mental impairments. The results provide strong evidence that UNICEF-supported initiatives together with previous large-scale trials demonstrate that nutrition-sensitive methods which include food fortification and social protection systems that use cash transfers can effectively decrease stunting and wasting. Multi-sectoral programs which focus on sanitation and infection control together with caregiver education provide more lasting advantages than single-component interventions which show that social factors beyond food access play a role in childhood malnutrition.

Children around the world still experience micronutrient deficiencies which particularly include iron deficiency anemia. The supplementation trials which researchers examined showed that hemoglobin levels and immune function and developmental outcomes all improved through the tested interventions which earlier meta-analyses identified as one of the most cost-effective public health interventions for iron supplementation. The vitamin A and iodine programs showed improved growth results which reduced illness rates in accordance with World Health Organization (WHO) guidelines that have existed for a long time. The common results which researchers found in different locations show that routine micronutrient programs need to continue because they protect vulnerable groups who include refugees and children from rural areas without resources.

Maternal and early childhood nutrition interventions show that malnutrition affects multiple generations of people. Evidence shows that better maternal nutrition during pregnancy and lactation leads to improved birth results and enhanced infant nutrient levels and better initial growth development. The results of this study support the developmental origins of health and disease (DOHaD) framework which states that early life experiences determine the risk of developing health problems in later life. Previous cohort studies have shown that maternal deficiencies lead to increased risk of low birth weight and skeletal development problems and subsequent anemia. The current review expands existing knowledge by demonstrating that maternal-child health programs which combine nutrition education with psychosocial support and water sanitation and hygiene (WASH) programs deliver better child health results both in the short term and long term.

The review contains long-term observational studies and cohort studies which show how nutritional deficiencies continue to affect individuals after their early childhood period. Children who experienced severe malnutrition during their early years faced ongoing difficulties with their growth patterns and development of anemia and micronutrient deficiencies throughout their subsequent growth periods. The findings of this study support earlier longitudinal studies which demonstrated that early nutritional deprivation leads to educational and metabolic and productivity impairments that endure throughout life. The observation results from different datasets show that child nutrition patterns have become more consistently tracked because of better global system monitoring and public health reporting, which leads to greater awareness about child nutrition patterns.

The study results show positive outcomes, but researchers need to examine specific methodological aspects. The evidence of publication bias which exists in multiple intervention-focused groups shows that smaller studies with null findings may be underrepresented according to previous research, which the previous nutrition meta-analyses confirmed. The study results display different research results because of variations in participant nutritional conditions and local feeding customs and medical facilities and study methods. The observed differences demonstrate that local communities need to develop their unique nutrition plans instead of following standardized models which exist throughout the world.

The evidence quality assessment showed evidence quality at three different levels because some observational studies had indirect evidence and their results were not precise enough. The low bias risk found in recent random control trials increases trust in the efficacy of both supplementation treatments and integrated intervention programs. Modern studies show better research methods than previous decades because current trials achieve higher standards which support evidence-based nutrition policies.

Public health experts need to establish multiple prevention programs which combine various approaches to stop childhood malnutrition and rickets and anemia. Organizations should implement nutrition-specific solutions together with their existing programs for poverty reduction and maternal education and food system resilience and equal healthcare distribution. The detection and prevention processes will receive better support through school-based supplementation and antenatal nutrition programs and community health worker programs.

### 4.2 Strengths

- The research study provides an extensive compilation of 96 studies which examine multiple geographic locations and different income levels from low-income to middle-income and high-income countries.
- The research study combines different study methods which include randomized controlled trials and observational studies and cohort analyses and meta-analyses.
- The research study includes multiple types of interventions which include micronutrient supplementation and maternal and child nutrition programs and integrated health and hygiene and behavior-change strategies.
- The researchers used standardized quality assessment tools together with risk-of-bias evaluation methods and publication bias analysis techniques to establish their methodological framework.
- The research team used subgroup analysis methods to study nutritional deficiencies and the impact of interventions throughout different population groups and stages of human development.

### 4.3 Limitations

- The research shows that intervention-oriented groups face publication bias which leads to their underreporting of non-significant results.
- Two types of observational studies experienced two problems, which led to their evidence base becoming less reliable.
- The different diagnostic criteria used to identify malnutrition, rickets, and anemia created inconsistencies in measurement results.
- The requirement to use only published literature causes researchers to miss important gray literature and program-level information from low-resource environments.
- Non-English studies were excluded.

### 4.4 Future prospects

- Researchers should perform extensive longitudinal studies which require proper design to study the extended impacts of early-life nutritional deficiencies.
- Researchers should establish standardized outcome measurements together with diagnostic standards which will assist in better cross-study research comparisons.
- The research should concentrate on developing practical nutrition solutions which provide affordable nutritional programs that can be implemented in different local environments.
- Public health strategies should include nutrition programs which combine maternal education efforts with food system improvements and WASH programs.
- The study will investigate new methods which include precision nutrition and digital health monitoring and community-based delivery systems to achieve early detection and targeted supplementation and worldwide monitoring.

## 5 Conclusion

The compiled research from 96 studies through this systematic review and meta-analysis reveals the various causes of childhood malnutrition, rickets, and anemia in different global settings. Vitamin D and calcium deficiencies are still the main reasons for nutritional rickets while deficiencies in micronutrients such as iron, vitamin A, and iodine are responsible for eventually impaired growth, cognitive, and immune abilities. Nutritional interventions for mothers and infants, particularly when combined with health, hygiene, and behavior-change strategies during critical periods like the first 1,000 days, always show protective effects. Long-term follow-up studies continuously show nutrition deficiency and thus underlining the necessity of constant monitoring and context-specific interventions. In general, the findings of this research support the very high position of comprehensive and multi-level nutrition programs which are directed not only toward children but also mothers, in the process of eliminating childhood malnutrition, preventing rickets and anemia, and ensuring long-term growth, development, and health outcomes.

## Data Availability

The raw data supporting the conclusions of this article will be made available by the authors, without undue reservation.
